# Association analysis of ILVBL gene polymorphisms with aspirin-exacerbated respiratory disease in asthma

**DOI:** 10.1186/s12890-017-0556-6

**Published:** 2017-12-16

**Authors:** Hun Soo Chang, Jong Sook Park, Ho Sung Lee, Jiwon Lyu, Ji-Hye Son, Inseon S. Choi, Hyoung Doo Shin, Choon-Sik Park

**Affiliations:** 1 Department of Medical Bioscience, Graduate School, Soonchunhyang University, 22, Soonchunhyang-ro, Asan, Chungcheongnam-do 336-745 Republic of Korea; 2 Division of Allergy and Respiratory Medicine, Department of Internal Medicine, Soonchunhyang University Bucheon Hospital, 1174, Jung-Dong, Wonmi-Ku, Bucheon, Gyeonggi-Do 420-021 Republic of Korea; 3 Division of Respiratory Medicine, Soonchunhyang University Chunan Hospital, Chunan-Si, Chungcheongnam-do 336-745 Republic of Korea; 4 Department of Allergy, Chonnam National University Medical School and Research Institute of Medical Sciences, Gwangju, 61469 Republic of Korea; 5 Department of Life Science, Sogang University, 1 Shinsu-dong, Mapo-gu, Seoul, 121-742 Republic of Korea; 6 Department of Genetic Epidemiology, SNP Genetics, Inc., 1407 14th Floor, Woolim-rall’ey B, Gasan-dong, Geumcheon-Gu, Seoul, 153-803 Republic of Korea

**Keywords:** AERD, ILVBL, Single nucleotide polymorphism, Association, Asthma

## Abstract

**Background:**

We previously reported that the *ILVBL* gene on chromosome 19p13.1 was associated with the risk for aspirin-exacerbated respiratory disease (AERD) and the percent decline of forced expired volume in one second (FEV1) after an oral aspirin challenge test. In this study, we confirmed the association between polymorphisms and haplotypes of the ILVBL gene and the risk for AERD and its phenotype.

**Methods:**

We recruited 141 AERD and 995 aspirin-tolerant asthmatic (ATA) subjects. All study subjects underwent an oral aspirin challenge (OAC). Nine single nucleotide polymorphisms (SNPs) with minor allele frequencies above 0.05, which were present in the region from 2 kb upstream to 0.5 kb downstream of ILVBL in Asian populations, were selected and genotyped.

**Results:**

In an allelic association analysis, seven of nine SNPs were significantly associated with the risk for AERD after correction for multiple comparisons. In a codominant model, the five SNPs making up block2 (*rs2240299*, *rs7507755*, *rs1468198*, *rs2074261*, and *rs13301*) showed significant associations with the risk for AERD (corrected *P* = 0.001–0.004, OR = 0.59–0.64). *Rs1468198* was also significantly associated with the percent decline in FEV1 in OAC tests after correction for multiple comparisons in the codominant model (corrected *P* = 0.033), but the other four SNPs in hapblock2 were not.

**Conclusion:**

To the best of our knowledge, this is the first report of an association between SNPs on *ILVBL* and AERD. SNPs on *ILVBL* could be promising genetic markers of this condition.

**Electronic supplementary material:**

The online version of this article (10.1186/s12890-017-0556-6) contains supplementary material, which is available to authorized users.

## Background

Aspirin (acetylsalicylic acid, ASA) hypersensitivity includes the ASA or other nonsteroidal anti-inflammatory drugs (NSAIDs)-induced respiratory disease of bronchoconstriction and nasal symptoms (AERD) and skin manifestations [1, 2]. The airway of AERD is characterized by infiltration of inflammatory cells and epithelial proliferation and disruption. Altered production of arachidonate metabolites by these cells account for the development of AERD [3].

Although AERD can be diagnosed with certainty by provocation tests, such as oral aspirin challenge (OAC) [4], OAC is a time-consuming procedure, and in some cases, serious complications can occur [2]. Thus, the development of noninvasive diagnostic methods such as the use of genetic marker sets is necessary to prevent the unexpected complications of aspirin use in susceptible patients. For the past two decades, many genetic association studies have demonstrated strong association of genetic variants on biologically plausible genes responsible for arachidonic acid metabolism, including *LTC4S* [5] *ALOX5* [6], *CYSLT1R* [7], *CYSLT2R* [8], *PTGER* [9–11], *TBXAS1* [12], and *TBXA2R* [13], with the development of AERD. Other studies also identified that genes in the immune response and inflammatory pathways were associated with the adverse reaction to aspirin,, including *HLA-DPB1* [14], *IL-4* [15], *T-Box* [16], *FcepsilonR1* [17, 18], *TLR3* [19], *NLRP3* [20], *ADAM33* [21], *ADORA1* [22], *ACE* [23], *CRTH2* [24], *PPARG* [25], *KIF3A* [26], *SLC6A12* [27], *SLC22A2* [28] and *CACNG6* [29]. These findings suggest that additional genetic variation in the extra-arachidonate pathways could be related to the development of AERD.

To identify a new genetic predisposition for the risk for AERD, we previously performed a genome-wide association study (GWAS) using a low-density 100 K [30] and a denser 660 K BeadChip [31]. On the basis of the 660 K GWAS study, which involved 430,486 single nucleotide polymorphisms (SNPs) in 802 asthmatics, a fine-mapping study of 702 SNPs on 14 genes was performed; the results showed significant associations with AERD in 1138 subjects. In that study, a nonsynonymous SNP in exon 2 of *HLA-DPB1*, *rs1042151* (Met105Val), showed the strongest association with the risk for AERD. In addition, the 660 K GWAS and fine-mapping studies revealed that the locus of *ILVBL* (IlvB (Bacterial Acetolactate Synthase)-Like) gene on chromosome 19p13.1 was associated with the risk for AERD and the percent decline of FEV1 after an OAC test.

*ILVBL* was first identified by Joutel et al. [32] from a human fetal brain cDNA library using a fragment isolated from a cosmid containing D19S841 at 19p13.1. They found that the 15-exon gene encodes a 632-amino-acid protein that shows similarity with several thiamine pyrophosphate-binding proteins identified in bacteria, yeast, and plants. Among them, the *ILVBL* gene showed the highest homologies with two bacterial enzymes, the B isozyme of the large catalytic subunit of *Escherichia coli* acetohydroxy-acid synthase (AHAS) and the oxalyl-CoA decarboxylase of *Oxalobacter formigenes*. Therefore, *ILVBL* is likely involved in branched-chain amino acid or pyruvate metabolism. Although a direct relationship between *ILVBL* or branched amino acid metabolism and aspirin or arachidonic acid metabolism has not been reported to date, our previous observation of an association between *ILVBL* polymorphisms and AERD suggests that this gene and its SNPs could be involved in the pathophysiology of the condition. In this study, we tried to confirm the allelic association of *ILVBL* gene in our previous study by analyzing associations of genotypes and haplotypes with the risk for AERD and with the percent decline of FEV1 as its phenotype.

## Methods

### Subjects

We recruited 141 AERD and 995 ATA Korean subjects from the Asthma Genome Research Center, which includes nine university hospitals in Korea. All patients were diagnosed by physicians and met the definition of asthma set forth in the Global Initiative for Asthma (GINA) guidelines [33]. Atopy was defined using skin-prick test for 24 common inhalant allergens as described in our previous report [31]. AERD and ATA were determined using an OAC test as described previously [34, 35]. The subjects in this study were identical with those in previous study [31] except two people who were failed to genotype ILVBL locus. All subjects provided informed written consent to participate in the study. All of the subjects provided written informed consent, and the protocol was approved by the Ethics Committee of Soonchunhyang University Hospital (approval No. SCHBC-IRB-2010-005).

### Genotyping

Twelve *ILVBL* polymorphisms were selected using the Asian population database from the International HapMap Project database (http://​hapmap.​ncbi.​nlm.​nih.​gov/​) and the NCBI database (http://​www.​ncbi.​nlm.​nih.​gov). SNP selection was based on the following scheme. First, candidate SNPs were extracted from the intragenic region including 2 kb of the 5′ region of each gene using Asian population data in the International HapMap database, and then LD structures of each gene were analyzed using SNPs with >5% minor allele frequencies. A representative of the SNPs in almost absolute LD (|D′| = 1 and *r* ^*2*^ > 0.95) was selected. A total of 702 SNPs were selected and genotyped using the GoldenGate assay with VeraCode microbeads (Illumina, Inc.) [36]. This was followed by scanning using the BeadXpress® system (Illumina, Inc.).

### Statistics

We used Lewontin’s D′ (|D′|) and r^2^ to measure linkage disequilibrium between biallelic loci [37]. The genotype and haplotype distributions were analyzed using logistic regression models with age (continuous value), gender (male = 0, female = 1), and smoking status (non-smoker = 0, ex-smoker = 1, smoker = 2) as covariates. Differences in the rates of decline in FEV1 following ASA challenge among the genotypes and haplotypes were examined using a type III generalized linear model. The data were managed and analyzed using SAS version 9.1 (SAS Inc., Cary, NC, USA), SPSS version 12.0 (SPSS Inc., Chicago, IL, USA) and PLINK version 1.9 (https://www.cog-genomics.org/plink2) [38]. For correction of *P*-values, the effective number of independent markers in *ILVBL* was calculated using the software SNPSpD (https://neurogenetics.qimrberghofer.edu.au/SNPSpD) [39]. The statistical power for the association analysis was calculated using Power for Genetic Association (PGA) version 2.0 [40]. The data are expressed as means ± standard errors of the mean (SE). P-values less than 5% were deemed to indicate statistical significance.

## Results

### Characteristics of the study subjects

In total, 1136 subjects were recruited from the asthma cohort, and their clinical characteristics are summarized in Table 1. AERD patients had a younger age of onset, higher proportion of smokers and nonsmokers, lower body mass index, and lower methacholine PC20 values than ATA patients. As expected, compared to ATA patients, the AERD subjects had a large percent decline of FEV1 after ASA challenge, a high ratio of patients with Water’s view, and a high neutrophil count in sputum (*P* < 0.05). Thus, age of onset, smoking status, and BMI, which were not related to AERD, were considered covariates in further analyses of genetic associations.

**Table 1:** Clinical characteristics of study subjects

	ATA	AERD	P
N	995	141	–
Sex (male, %)	38.5%	38.3%	0.965
Age (yr)	44.8 ± 0.49	42.39 ± 1.26	0.082
Age of onset (yr)	38.82 ± 0.53	34.5 ± 1.53	0.007
Smoking status (NS/ES/SM, %)	69.6/16.6/13.7	79.4/5.0/15.6	0.002
Body mass index (kg/m2)	24.38 ± 0.11	23.65 ± 0.3	0.023
FEV1 before ASA challenge (% predicted)	83.42 ± 0.63	80.52 ± 1.76	0.124
Decline of FEV1 after ASA challenge (%)	3.83 ± 0.16	32.42 ± 1.08	1.14× 10^−53^
log(PC20 methacholine (mg/mL))	0.36 ± 0.02	−0.02 ± 0.07	1.13× 10^−7^
Atopy (Y, %)	51.9%	48.2%	0.419
Serum total IgE (kU/L)	393.02 ± 20.22	411.2 ± 60.71	0.768
Urticaria (Y, %)	22.0%	19.9%	0.562
Water’s view (Y, %)	34.7%	59.6%	1.14× 10^−8^
Peripheral eosinophil count	119.88 ± 4.72	112.2 ± 13.97	0.603
Sputum eosinophil (%)	33.37 ± 1.26	32.65 ± 3.83	0.858
Sputum neutrophil (%)	5.66 ± 0.51	11.35 ± 2.31	0.018

*ATA* aspirin tolerant asthmatics, *AERD* aspirin-exacerbated respiratory disease, *NS* never smokers, *ES* ex-smokers, *SM* current smokers

Numeric data were presented as mean ± standard error

*P* values were obtained using independent t-test or χ^2^ test

### *Frequency*, *heterozygosity*, *and the Hardy–Weinberg equilibrium of SNPs in* ILVBL

**Fig. 1:**
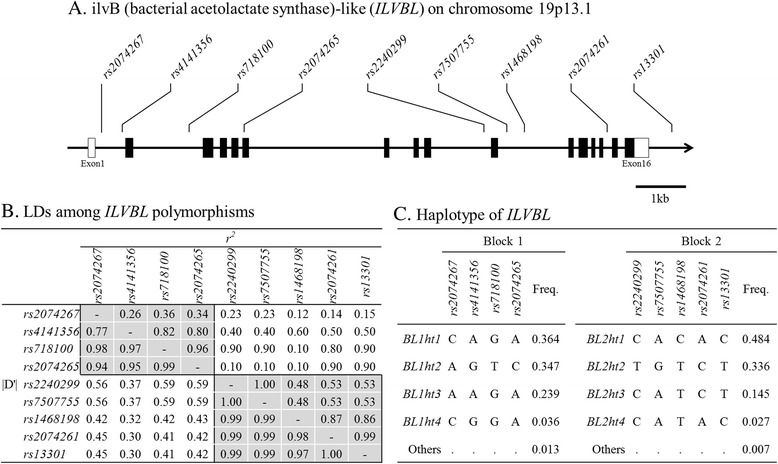
(**a**) Schematic gene map and SNPs in the *ILVBL* gene on chromosome 19p13.1. Black boxes represent coding exons and white boxes represent 5′ and 3’ UTRs. (**b**) Pairwise linkage disequilibrium among *ILVBL* SNPs. Shaded boxes represent haplotype blocks. (**c**) Haplotypes and their frequencies in each haplotype block

According to dbSNP (http://www.ncbi.nlm.nih.gov/SNP) and Hapmap DB (http://hapmap.ncbi.nlm.nih.gov), nine SNPs with minor allele frequencies above 0.05 are present in the region from 2 kb upstream to 0.5 kb downstream of ILVBL in Asian populations (Han Chinese and Japanese): *rs2074267*, *rs4141356*, *rs718100*, *rs2074265*, *rs2240299*, *rs7507755*, *rs1468198*, *rs2074261*, and *rs13301*. Among them, two were in the 5’-UTR (*rs2074267* and *rs4141356*), five were in the intronic sequences (*rs718100*, *rs2240299*, *rs7507755*, *rs1468198* and *rs2074261*), one was in the coding region (*rs2074265*, L213 L), and one was in the 3′ region downstream of the gene (*rs13301*). The gene map and location of the SNPs are presented in Fig. 1a.

The Hardy–Weinberg equilibrium of the nine SNPs are summarized in Additional file 1: Table S1. The distributions of all loci were in Hardy–Weinberg equilibrium in both AERD and ATA subjects (*P* > 0.01). The calculated linkage disequilibrium coefficients |D′| and *r* ^*2*^ among the SNPs revealed that *ILVBL* was parsed into two LD blocks (BLs) and that there were four major haplotypes (frequency > 0.01) for each of BL1 and BL2 (Fig. 1b-c). Among the four common haplotypes of BL1, haplotype1 (BL1ht2) was excluded from further statistical analysis because it was almost equivalent to *rs718100* and *rs2074265*. Similarly, only haplotype3 (BL2ht3) and haplotype4 (BL2ht4) were used for further statistical analysis because BL2ht1 was almost equivalent to *rs1468198*, and BL2ht2 was almost the same as *rs2240299* and *rs7507755*.

### *Associations between* ILVBL *polymorphisms and the risk for and phenotypes of AERD in asthmatics*

The *ILVBL* polymorphisms and haplotypes were analyzed in terms of their associations with the risk for AERD using multiple logistic regression models. In the allelic association analysis, seven of nine SNPs were significantly associated with the risk for AERD after correction for multiple comparisons (Table 2). The MAFs of *rs2074265* and *rs718100* in block1 tended to be higher in AERD with marginal *P* values (corrected *P* = 0.046–0.049). In contrast, the MAFs of *rs2240299*, *rs7507755*, *rs1468198*, *rs2074261*, and *rs13301*, which were in block2, were significantly lower in AERD than in ATA (corrected *P* = 0.001–0.003). In the codominant model, the five SNPs making up block2 showed significant associations with the risk for AERD (corrected P = 0.001–0.004, OR = 0.59–0.64; Table 3), but none in block1 were associated with AERD (corrected *P* > 0.05). Statistical powers for the association of *rs1468198* were 91.0%, 97.8% and 77.8% for codominant, dominant, and recessive model, respectively. Although the number of minor allele homozygotes on *rs2240299* and *rs7507755* was small (*n* = 7), the powers for other significant associations, including rs2240299 and rs7507755, were between 82.1% and 89.8%.

**Table 2:** Comparison of minor allele and haplotype frequencies in *ILVBL* gene with the risk of AERD

Locus	Allele	Location	MAF		OR [95% CI]	P^*^	P_corr_^**^
			AERD	ATA			
*rs2074267*	*C > A*	5’-UTR	0.358	0.414	0.79 [0.61–1.03]	0.076	0.699
*rs4141356*	*G > A*	5’-UTR	0.447	0.378	1.33 [1.03–1.71]	0.027	0.250
*rs718100*	*T > G*	intron 2	0.429	0.344	1.44 [1.11–1.85]	0.005	0.046
*rs2074265*	*C > A*	Exon 6 (L213 L)	0.433	0.348	1.43 [1.11–1.84]	0.005	0.049
*rs2240299*	*T > C*	intron 9	0.241	0.352	0.58 [0.44–0.78]	2.28 × 10^−4^	0.002
*rs7507755*	*G > A*	intron 10	0.241	0.352	0.59 [0.44–0.78]	2.38 × 10^−4^	0.002
*rs1468198*	*C > T*	intron 10	0.475	0.603	0.60 [0.46–0.77]	6.11 × 10^−5^	0.001
*rs2074261*	*C > A*	intron 14	0.379	0.501	0.61 [0.47–0.79]	1.41 × 10^−4^	0.001
*rs13301*	*T > C*	3′-flanking	0.387	0.502	0.63 [0.48–0.81]	2.82 × 10^−4^	0.003
*BL1ht1*	*CAGA*	–	0.316	0.371	0.78 [0.60–1.02]	0.072	0.580
*BL1ht3*	*AAGA*	–	0.220	0.242	0.88 [0.65–1.19]	0.410	1.000
*BL1ht4*	*CGGA*	–	0.032	0.037	0.86 [0.43–1.75]	0.684	1.000
*BL2ht3*	*CATCT*	–	0.135	0.146	0.91 [0.63–1.31]	0.608	1.000
*BL2ht4*	*CATAC*	–	0.021	0.028	0.75 [0.32–1.76]	0.508	1.000

*MAF* minor allele frequency, *ATA* aspirin tolerant asthmatics, *AERD* aspirin-exacerbated respiratory disease, *OR* odd ratio, *CI* confidence interval

^*^ *P* values were obtained using logistic regression analysis controlling age of onset, smoking status and BMI as covariates

^**^Corrected P values for multiple comparison using SNPSpD

**Table 3:** Genotype and haplotype association analysis in *ILVBL* gene with the risk of AERD

Locus	Diag	Genotype				Codominant.			Dominant.			Recessive		
		RR	CR	CC	Total	OR [95% CI]	P^*^	P_corr_^**^	OR [95% CI]	P^*^	P_corr_^**^	OR [95% CI]	P^*^	P_corr_^**^
*rs2074267*	AERD	20 (14.2%)	61 (43.3%)	60 (42.6%)	141 (100%)	0.79 [0.61–1.04]	0.087	0.799	0.67 [0.47–0.96]	0.030	0.273	0.91 [0.55–1.52]	0.729	1.000
	ATA	153 (15.4%)	517 (52.0%)	325 (32.7%)	995 (100%)									
*rs4141356*	AERD	31 (22.0%)	64 (45.4%)	46 (32.6%)	141 (100%)	1.33 [1.04–1.72]	0.026	0.235	1.3 [0.89–1.89]	0.174	1.000	1.73 [1.12–2.69]	0.014	0.131
	ATA	140 (14.1%)	473 (47.5%)	382 (38.4%)	995 (100%)									
*rs718100*	AERD	30 (21.3%)	61 (43.3%)	50 (35.5%)	141 (100%)	1.43 [1.11–1.85]	0.006	0.050	1.38 [0.95–1.99]	0.089	0.819	2.04 [1.30–3.20]	0.002	0.017
	ATA	116 (11.7%)	452 (45.4%)	427 (42.9%)	995 (100%)									
*rs2074265*	AERD	30 (21.3%)	62 (44.0%)	49 (34.8%)	141 (100%)	1.43 [1.11–1.84]	0.006	0.055	1.39 [0.96–2.01]	0.081	0.745	1.98 [1.27–3.11]	0.003	0.026
	ATA	119 (12.0%)	454 (45.6%)	422 (42.4%)	995 (100%)									
*rs2240299*	AERD	7 (5.0%)	54 (38.3%)	80 (56.7%)	141 (100%)	0.59 [0.45–0.79]	3.65 × 10^−4^	0.003	0.57 [0.4–0.82]	0.002	0.020	0.34 [0.16–0.75]	0.007	0.068
	ATA	131 (13.2%)	438 (44.1%)	425 (42.8%)	994 (100%)									
*rs7507755*	AERD	7 (5.0%)	54 (38.3%)	80 (56.7%)	141 (100%)	0.59 [0.45–0.79]	3.78 × 10^−4^	0.003	0.57 [0.4–0.82]	0.002	0.021	0.34 [0.16–0.75]	0.008	0.069
	ATA	131 (13.2%)	438 (44.0%)	426 (42.8%)	995 (100%)									
*rs1468198*	AERD	22 (15.6%)	68 (48.2%)	51 (36.2%)	141 (100%)	0.60 [0.47–0.78]	9.53 × 10^−4^	0.001	0.47 [0.29–0.75]	0.002	0.016	0.53 [0.37–0.78]	0.001	0.010
	ATA	279 (28.1%)	483 (48.7%)	230 (23.2%)	992 (100%)									
*rs2074261*	AERD	22 (15.6%)	63 (44.7%)	56 (39.7%)	141 (100%)	0.62 [0.48–0.8]	2.30 × 10^−4^	0.002	0.54 [0.38–0.78]	0.001	0.010	0.51 [0.31–0.82]	0.005	0.048
	ATA	263 (26.5%)	469 (47.2%)	262 (26.4%)	994 (100%)									
*rs13301*	AERD	22 (15.6%)	65 (46.1%)	54 (38.3%)	141 (100%)	0.64 [0.5–0.82]	4.20 × 10^−4^	0.004	0.57 [0.39–0.83]	0.003	0.027	0.5 [0.31–0.81]	0.005	0.043
	ATA	265 (26.7%)	468 (47.1%)	261 (26.3%)	994 (100%)									
		Haplotype												
		+/+	−/+	−/−	Total									
*BL1ht1*	AERD	16 (11.3%)	57 (40.4%)	68 (48.2%)	141 (100%)	0.78 [0.6–1.03]	0.084	0.672	0.67 [0.47–0.96]	0.028	0.226	0.94 [0.54–1.64]	0.829	1.000
	ATA	119 (12.0%)	498 (50.2%)	376 (37.9%)	993 (100%)									
*BL1ht3*	AERD	9 (6.4%)	44 (31.2%)	88 (62.4%)	141 (100%)	0.86 [0.64–1.17]	0.343	1.000	0.79 [0.55–1.14]	0.213	1.000	1.06 [0.51–2.21]	0.873	1.000
	ATA	57 (5.7%)	367 (37.0%)	569 (57.3%)	993 (100%)									
*BL1ht4*	AERD	0 (0.0%)	9 (6.4%)	132 (93.6%)	141 (100%)	0.86 [0.42–1.76]	0.688	1.000	0.87 [0.42–1.79]	0.709	1.000	inf	0.999	1.000
	ATA	1 (0.1%)	71 (7.2%)	921 (92.7%)	993 (100%)									
*BL2ht3*	AERD	3 (2.1%)	32 (22.7%)	106 (75.2%)	141 (100%)	0.9 [0.62–1.3]	0.565	1.000	0.86 [0.57–1.3]	0.486	1.000	1.12 [0.33–3.85]	0.856	1.000
	ATA	19 (1.9%)	253 (25.4%)	723 (72.7%)	995 (100%)									
*BL2ht4*	AERD	0 (0%)	6 (4.3%)	135 (95.7%)	141 (100%)	0.77 [0.33–1.8]	0.541	1.000	0.77 [0.33–1.84]	0.560	1.000	inf	0.999	1.000
	ATA	1 (0.1%)	54 (5.4%)	940 (94.5%)	995 (100%)									

*R* rare allele, *C* common allele, *ATA* aspirin tolerant asthmatics, *AERD* aspirin-exacerbated respiratory disease, *OR* odd ratio, *CI* confidence interval

*P values were obtained using logistic regression analysis controlling age of onset, smoking status and BMI as covariates

**Corrected *P* values for multiple comparison using SNPSpD

Because ASA-induced decline in FEV_1_ is the most important parameter for the diagnosis of ASA intolerance in asthmatics, we tested the associations between SNPs and haplotypes and the rate of decline in FEV_1_ following ASA challenge (Table 4). Among the nine SNPs, *rs1468198* showed a significant association with the percent decline in FEV1 in OAC tests after correction for multiple comparisons in the codominant model (corrected *P* = 0.033). Common allele homozygotes showed a greater percent decline than did minor allele homozygotes (8.02 ± 0.76 vs. 5.67 ± 0.54). The other four SNPs in hapblock2 also showed significant associations with the percent reduction of FEV1; however, these were not statistically significant after correction for multiple comparisons. None of the common haplotypes showed an association with the percent reduction of FEV1 by OAC. The results of covariate-unadjusted models of the analyses using independent *t*-test and one-way ANOVA were similar; only *rs1468198* showed a significant association with the percent reduction of FEV1 in the codominant model (corrected *P* = 0.034; data not shown).

**Table 4:** Genotype and haplotype association analysis in *ILVBL* gene with % decline of FEV1 after oral aspirin challenge test in asthmatics

Locus	Genotype			Codominant		Dominant		Recessive	
	RR	CR	CC	P^*^	P_corr_^**^	P^*^	P_corr_^**^	P^*^	P_corr_^**^
*rs2074267*	6.95 ± 0.76 (173)	6.29 ± 0.46 (578)	7.18 ± 0.59 (385)	0.413	1.000	0.207	1.000	0.907	1.000
*rs4141356*	7.53 ± 0.90 (171)	6.57 ± 0.48 (537)	6.51 ± 0.51 (428)	0.271	1.000	0.484	1.000	0.238	1.000
*rs718100*	8.27 ± 1.04 (146)	6.36 ± 0.49 (513)	6.56 ± 0.48 (477)	0.138	1.000	0.500	1.000	0.043	0.391
*rs2074265*	8.15 ± 1.02 (149)	6.46 ± 0.49 (516)	6.48 ± 0.48 (471)	0.120	1.000	0.389	1.000	0.058	0.535
*rs2240299*	4.99 ± 0.61 (138)	6.33 ± 0.49 (492)	7.51 ± 0.53 (505)	0.006	0.053	0.015	0.139	0.041	0.376
*rs7507755*	4.99 ± 0.61 (138)	6.33 ± 0.49 (492)	7.50 ± 0.53 (506)	0.006	0.055	0.016	0.145	0.041	0.379
*rs1468198*	5.67 ± 0.54 (301)	6.59 ± 0.47 (551)	8.02 ± 0.76 (281)	0.004	0.033	0.008	0.072	0.033	0.307
*rs2074261*	5.76 ± 0.57 (285)	6.47 ± 0.46 (532)	7.88 ± 0.71 (318)	0.006	0.057	0.009	0.078	0.061	0.560
*rs13301*	5.78 ± 0.57 (287)	6.62 ± 0.47 (533)	7.63 ± 0.69 (315)	0.015	0.139	0.032	0.291	0.064	0.590
	Haplotype								
	+/+	−/+	−/−						
*BL1ht1*	7.25 ± 0.55 (444)	6.22 ± 0.46 (555)	6.78 ± 0.82 (135)	0.276	1.000	0.151	1.000	0.949	1.000
*BL1ht3*	6.89 ± 0.44 (657)	6.22 ± 0.52 (411)	7.58 ± 1.51 (66)	0.693	1.000	0.563	1.000	0.845	1.000
*BL1ht4*	6.66 ± 0.34 (1053)	7.16 ± 1.24 (80)	3.00 (1)	0.964	1.000	0.943	1.000	0.827	1.000
*BL2ht3*	6.70 ± 0.38 (829)	6.65 ± 0.67 (285)	6.91 ± 1.77 (22)	0.818	1.000	0.752	1.000	0.845	1.000
*BL2ht4*	6.72 ± 0.34 (1075)	6.21 ± 1.39 (60)	0.00 (1)	0.564	1.000	0.612	1.000	0.524	1.000

*R* rare allele, *C* common allele

*P values were obtained using linear regression analysis controlling age of onset, smoking status and BMI as covariates

**Corrected *P* values for multiple comparison using SNPSpD

### *Association analysis using* rs1468198 *as a covariate*

SNPs in block2 were in high linkage disequilibrium (|D’| > 0.97 and *r* ^*2*^ > 0.5; Fig. 1b). To evaluate possible causative SNPs in the block independent of *rs1468198*, which was the most significant SNP, we tested the association between genotypes and AERD and percent decline of FEV1 using *rs1468198* as a covariate, together with age of onset, smoking status, and BMI. No SNP other than *rs1468198* showed an association with AERD or percent decline of FEV1 (Table 5). These results indicate that the observed associations between other SNPs in block2 and AERD were based on their tight LD with *rs1468198*.

**Table 5:** The association of SNPs in ILVBL gene with the risk of AERD and % decline of FEV1 after adjusting *rs1468198*

The risk of AERD			
SNP	OR [95% CI]	P^*^	P_corr_^†^
*rs13301*	1.16 [0.55–2.41]	0.699	1.000
*rs2074261*	0.99 [0.47–2.09]	0.975	1.000
*rs7507755*	0.78 [0.52–1.17]	0.226	1.000
*rs2240299*	0.78 [0.52–1.16]	0.220	1.000
*rs2074265*	1.24 [0.94–1.61]	0.123	1.000
*rs718100*	1.24 [0.95–1.63]	0.109	1.000
*rs4141356*	1.18 [0.91–1.53]	0.222	1.000
*rs2074267*	0.96 [0.72–1.28]	0.772	1.000
% decline of FEV1			
SNP	β (± SE)	P^**^	P_corr_^†^
*rs13301*	1.08 ± 1.25	0.387	1.000
*rs2074261*	0.06 ± 1.29	0.964	1.000
*rs7507755*	−0.64 ± 0.69	0.353	1.000
*rs2240299*	−0.65 ± 0.69	0.344	1.000
*rs2074265*	0.34 ± 0.51	0.505	1.000
*rs718100*	0.31 ± 0.51	0.535	1.000
*rs4141356*	0.19 ± 0.49	0.702	1.000
*rs2074267*	0.12 ± 0.52	0.814	1.000

*, ** P values were obtained using logistic and linear regression analysis, respectively, controlling age of onset, smoking status, BMI, and genotype of *rs1468198*as covariates

† Corrected P values for multiple comparison using SNPSpD

*OR* odd ratio, *CI* confidence interval, *SE* standard error

## Discussion

Based on the results of our previous GWAS study for AERD, we evaluated the associations between *ILVBL* polymorphisms and the risk for AERD and percent decline of FEV1 after OAC tests in subjects with asthma. In our previous GWAS, among three SNPs in *ILVBL* on the 660 W BeadChip, *rs2240299*, an intronic SNP in *ILVBL*, showed a significant association with the risk for AERD (odds ratio = 0.51 [0.37–0.72], *P* = 7.61 × 10^−5^) and the percent decline of FEV1 after an OAC test (*P* = 0.004). In the present fine genotyping and association study for validation, among nine SNPs in the ILVBL gene, *rs1468198* and SNPs linked with *rs1468198* showed significant associations with the phenotypes of AERD. Although regarding multiple comparison derived by three genetic model and two outcome testing, the association between *rs1468198* and the risk of AERD were statistically significant (SNPSpD corrected P × 3 genetic models × 2 phentoypes = 0.006). Our observations suggest that the *ILVBL* gene and its locus play a role in the pathogenesis of AERD. To the best of our knowledge, there is no previous report of a genetic association with AERD, or any other disease.

Aspirin has antipyretic, anti-inflammatory, analgesic, and antiplatelet effects by irreversible inhibition of cyclooxygenase-1 (COX-1) and regulation of various receptors and signaling molecules. The analgesic effects of non-steroidal anti-inflammatory drugs (NSAID) are mediated by beta2 adrenergic receptors (β2ADR). Suleyman et al. [41] and Caidrci et al. [42] independently revealed that the analgesic and anti-inflammatory effects of NSAIDs including aspirin were lost in adrenalectomized rats compared to normal rats. The analgesic and anti-inflammatory effects of NSAIDs were restored by pretreatment of rats with prednisolone and adrenalin, an effect which was inhibited by beta2 receptor antagonists but not by alpha1, alpha2, or beta1 antagonists [41, 42]. Moreover, polymorphisms in the β2ADR were associated with AERD and with aspirin-intolerant acute urticaria [43, 44]. In addition, aspirin and its derivatives prevent cancer cell proliferation by reducing epidermal growth factor receptor (EGFR) expression and downstream signal transduction [45–47]. The therapeutic/chemopreventative effects of aspirin in cancer are also mediated by direct inhibition of integrin-linked kinase (ILK) signaling and by decreased expression of c-Myc in cancer cells [48–51].

Due to its high structural similarity with bacterial acetolactate synthases, *ILVBL* has been postulated to be involved in pyruvate or branched amino acid metabolism, but the precise function of the gene product is unclear. However, recent proteomic studies have revealed that the ILVBL protein interacts with various factors, including β2ADR, EGFR, ILK, and c-MYC [52–55]. Therefore, the *ILVBL* gene could be involved in the functions and regulation of these proteins, which are related to the mechanism of action of aspirin. Thus, the roles and functions of *ILVBL* and its interacting proteins in the pathophysiology of aspirin hypersensitivity warrant further studies.

In this study, *rs1468198* located on the 10th intron of *ILVBL* and SNPs on the same hapblock with *rs1468198* showed significant associations with AERD phenotype. After adjusting for *rs1468198*, the remaining SNPs showed no significant association, which suggests that *rs1468198* is the most promising causative polymorphism for AERD. With the exception of a report of an association between copy number variation in the region including *ILVBL* and the pathogenesis of seizure, intrauterine growth retardation, learning disability, microcephaly, and intellectual disability [56], there has been no report of associations between SNPs in *ILVBL* and disease. According to functional estimation of the SNPs linked with *rs1468198* in Asian populations (SNPinfo Web Server, https://snpinfo.niehs.nih.gov/), *rs1468198* did not show transcription factor binding, splicing site, splicing regulation, or miRNA molecular functions. Instead, *rs2074262*, which was not included in this study but which is located 1208 bp downstream of *rs1468198* and 359 bp upstream of *rs2074261*, is located on a splicing enhancer and is highly conserved. This in silico prediction suggests that the observed association between *rs1468198* and AERD could be due to its high LD with *rs2074262*, which could affect post-transcriptional processing of *ILVBL*. In addition, although it was not statistically significant after correction of multiple comparison, *rs2074262* showed a trend of association with mRNA expression of RAR related orphan receptor A (RORA, *P* = 0.00007) and somatostatin receptor 3 (SSTR3, *P* = 0.0001) gene in expression quatitative trait loci (eQTL) analysis using ENCODE dataset (https://www.encodeproject.org). These genes may be involved in asthma- and AERD-related cytokine signaling such as IL-4 and IL-13 (http://reactome.org). Thus, the roles of *ILVBL*, as well as the functional consequences of *rs1468198* and *rs2074262*, in the pathophysiology of AERD should be evaluated in further study.

The present study has several limitations. Firstly, only nine SNPs in the ILVBL gene were evaluated in this study. In the ILVBL gene, which is spanning 11 kb of chromosome 19p13.1, 1548 SNPs registered in dbSNP (http://www.ncbi.nlm.nih.gov/snp), including 29 SNPs with MAF > 0.05. Although the SNPs analyzed in this study tagged haplotypes on each hapblock (Fig. 1c), the other SNPs may be directly associated with AERD itself or related phenotypes. This possibility should be confirmed in further replication studies that include high-density markers with low frequencies and use next-generation sequencing or exome variant analyses. In addition, population stratification bias can be introduced in genetic association studies [57]. However, we consider such a bias to be unlikely because the Korean population is reported to show a relatively high degree of genetic homogeneity [58].

## Conclusions

We found a significant association between polymorphisms of *ILVBL*, a candidate gene in patients with AERD, and the risk for and phenotypes of AERD in patients with asthma. Further investigations of the biological roles of the ILVBL protein in the mechanism of action of aspirin and in the pathogenesis of AERD should be performed, particularly regarding its interactions with other proteins. To the best of our knowledge, this is the first report of an association between SNPs on *ILVBL* and AERD. Our results also suggest that SNPs on *ILVBL* are potential genetic markers for AERD.

## Additional file

Additional file 1: Table S1. Hardy-Weinberg Equilibrium of SNPs and haplotypes in ILVBL gene. (XLS 27 kb)

## Abbreviations

AERD: Aspirin-exacerbated respiratory disease
AHAS: Acetohydroxy-acid synthase
ASA: Acetylsalicylic acid, aspirin
ATA: Aspirin-tolerant asthma
BL: LD block
BMI: Body mass index
eQTL: Expression quatitative trait loci
FEV1: Forced expired volume in one second
GINA: Global Initiative for Asthma
GWAS: Genome-wide association study
Ht: Haplotype
LD: Linkage Disequilibrium
NSAID: Nonsteroidal anti-inflammatory drug
OAC: Oral aspirin challenge
PC20: Provocative concentration of methacholine causing a 20% fall in FEV1
PGA: Power for Genetic Association
SE: Standard errors of the mean
SNP: Single nucleotide polymorphism
UTR: Untranslated region
